# Growth Performance and Postprandial Metabolic Routing in Red Abalone (*Haliotis rufescens*): Comparing *Bacillus*-Supplemented Plant- and Fishmeal-Based Diets to Fresh *Macrocystis pyrifera*

**DOI:** 10.3390/ani16132048

**Published:** 2026-07-03

**Authors:** Jorge Olmos, Manuel Acosta, Jeremie Bauer, Fernando Díaz, Denisse Re-Araujo, Juan Pablo Sánchez-Ovando, Melany Sánchez-González, Alfonso Rodríguez

**Affiliations:** Marine Biotechnology Department, Centro de Investigación Científica y Educación Superior de Ensenada (CICESE), Carretera Tijuana-Ensenada 3918, Fraccionamiento Playitas, Ensenada 22860, Mexico; macosta@cicese.edu.mx (M.A.); jbauer@cicese.edu.mx (J.B.); fdiaz@cicese.edu.mx (F.D.); denisre@cicese.edu.mx (D.R.-A.); sanchezjp@cicese.edu.mx (J.P.S.-O.); melany@cicese.edu.mx (M.S.-G.); alfonso.rodriguez@cicese.edu.mx (A.R.)

**Keywords:** plant-based ingredients, *Bacillus* probiotics, specific dynamic action, nutrient bioavailability, functional feeds, sustainable aquafeed

## Abstract

Plant-based formulations supplemented with probiotics are a promising strategy to improve nutrient bioavailability in aquaculture, yet mechanistic understanding of microbial modulation of host metabolism remains limited. We evaluated whether supplementation with *Bacillus velezensis* (Sp1) and *B. amyloliquefaciens* (Sp3) influences metabolic efficiency and energy allocation in sub-adult red abalone (*Haliotis rufescens*). Over 180 days, animals were fed a plant-based or a fishmeal-based diet—both probiotic-supplemented and matched in protein and energy—and compared with fresh kelp (*Macrocystis pyrifera*) as a control. Metabolic performance was assessed from oxygen consumption, relative specific dynamic action (SDA) index, ammonia excretion and Oxygen-to-nitrogen ratio (O:N) ratio. The *Bacillus*-supplemented plant-based diet achieved metabolic efficiency comparable to *M. pyrifera*, with minimal SDA (4.7% vs. 4.3%) and commercially viable growth (2.07 mm/month). The O:N ratio indicated diet-specific, time-dependent shifts in substrate use. The fishmeal-based diet—despite identical macronutrient and probiotic composition—produced a markedly higher postprandial metabolic cost (SDA = 21.0%), consistent with reduced compatibility between high animal protein and herbivorous digestive physiology. Overall, probiotic supplementation may help convert plant-based ingredients into metabolically efficient functional feeds, offering a more sustainable approach that reduces dependence on marine-derived ingredients.

## Simple Summary

Red abalone is a valuable shellfish raised on farms, but feeding it sustainably is a challenge. Farmers traditionally rely on fresh giant kelp, a seaweed that is increasingly scarce, costly to harvest, and disappearing from coastal waters. Manufactured feeds based on fishmeal offer an alternative, yet they are expensive and place pressure on wild fish stocks. This study asked whether a plant-based feed enriched with helpful bacteria could nourish farmed abalone as effectively as fresh seaweed. Over six months, we compared three diets: fresh kelp, a plant-based feed with added bacteria, and a fishmeal-based feed with the same bacteria. The plant-based feed produced healthy growth close to that of kelp and encouraged better reproductive development. The fishmeal feed performed less well, possibly because the animals processed its rich protein less efficiently. These findings suggest that helpful bacteria may help abalone to digest affordable plant ingredients more efficiently. For society, this potentially offers a cheaper, more sustainable way to farm abalone while easing the strain on wild macroalgae and ocean fish, supporting both coastal livelihoods and marine conservation.

## 1. Introduction

Red abalone (*Haliotis rufescens*) is a commercially valuable gastropod cultivated along the Northeastern Pacific coast [1]. Despite substantial market demand and favorable biological characteristics, including rapid growth and high value, *H. rufescens* aquaculture expansion faces constraints related to feed costs, availability, and nutritional [2]. In Mexico, fresh giant kelp (*Macrocystis pyrifera*) has historically served as the primary feed source for abalone cultivation due to its natural role in abalone ecology, availability and demonstrated growth performance [3,4]. However, dependence on wild kelp harvesting introduces operational vulnerabilities, including seasonal availability fluctuations, variability in nutritional quality, collection and transport logistics, and wild population decline [5,6,7,8]. These constraints demand the development of cost-effective, nutritionally balanced formulated feeds that can support sustainable abalone production while reducing dependence on wild-harvested resources.

Formulated feed development for abalone has traditionally relied on fishmeal due to its high protein content (60–70%), amino acid profile, and established use in aquafeeds [2,9,10]. However, fishmeal-based diets present multiple limitations that constrain their application. Global fishmeal supplies face increasing pressure from demand across multiple aquaculture sectors, driving substantial price volatility and raising sustainability concerns regarding capture fisheries [11,12]. Moreover, the metabolic inefficiencies associated with high fishmeal inclusion, including elevated nitrogen excretion and reduced feed conversion efficiency, further limit the cost-effectiveness of fishmeal-based formulations [13]. In that sense, plant-based protein alternatives—including soybean meal and other terrestrial plant sources—which offer economic advantages, sustainable production systems, and potentially superior digestibility in abalone, are being explored [14,15,16].

Plant-based ingredients, while economically attractive and environmentally sustainable, present distinct challenges related to nutrient bioavailability, the presence of anti-nutritional factors (ANFs), and variable digestibility [17]. Probiotic supplementation has emerged as a strategy to enhance nutrient utilization in formulated aquafeeds through production of degradative enzymes, improvement of the gut microbiome, enhancement of immune function, and reduction of pathogenic bacteria in aquaculture species [18,19,20,21]. Among probiotics, *Bacillus* species have demonstrated beneficial effects in various aquaculture species due to their capacity to produce carbohydrase, protease and lipase enzymes, their thermostability during feed processing, and their classification as Generally Recognized as Safe (GRAS) [19,20]. In abalone specifically, probiotic manipulation of the gut microbiome has shown promising effects on both somatic growth and reproductive development [22,23,24,25]. Nonetheless, mechanisms by which probiotics influence metabolic efficiency and energy allocation in abalone fed plant-based diets require investigation to optimize formulation strategies.

Measuring metabolic changes that occur after feeding is crucial for evaluating the quality of consumed feed and elucidating mechanisms by which organisms allocate available energy [26]. Oxygen consumption rates reflect the metabolic demands of processing feed ingredients through digestion and nutrient absorption, with the energetic cost of these postprandial processes being quantified as specific dynamic action (SDA) [27]. Ammonia excretion patterns indicate nitrogen utilization efficiency, with elevated excretion signaling poor protein retention or excessive protein catabolism for energy rather than tissue synthesis [28]. The oxygen:nitrogen ratio (O:N) provides insight into metabolic substrate preferences, revealing whether organisms primarily catabolize proteins, carbohydrates, or lipids to meet energetic demands [29,30]. Integration of these metabolic parameters with growth performance data enables mechanistic understanding of diet-dependent differences in energy partitioning between somatic growth, maintenance metabolism, and reproductive investment [27]. In abalone, metabolic characterization has revealed fundamental aspects of nutritional biochemistry, including predominant reliance on protein and carbohydrate catabolism, minimal lipid metabolism, and ontogenetic shifts in metabolic rate and substrate preferences [30,31,32]. However, comprehensive metabolic assessment of plant-based diets supplemented with probiotic strains in sub-adult abalone remains poorly studied.

The present study addresses these knowledge gaps through the assessment of growth performance and postprandial metabolism in sub-adult *H. rufescens* fed three dietary treatments: (1) a plant-based formulated diet supplemented with two probiotic *Bacillus* strains, (2) a fishmeal-based formulated diet with the same probiotic supplementation and macronutrient composition, and (3) fresh *M. pyrifera* as a natural diet control used in commercial aquaculture. Specifically, we evaluate whether a plant-based diet supplemented with probiotics can achieve growth performance and metabolic efficiency comparable to fresh kelp. We employ metabolic profiling, including oxygen consumption rate, to determine SDA, ammonia excretion rate, and O:N ratio, and to elucidate mechanistic bases for diet-dependent differences in energy allocation. These findings aim to inform sustainable feed development strategies for abalone aquaculture that reduce dependence on both wild kelp harvesting and fishmeal while supporting production objectives ranging from market-size animal cultivation to broodstock conditioning.

## 2. Materials and Methods

### 2.1. Experimental Design and Animal Husbandry

A 180-day feeding trial was conducted from 25 October 2024 to 23 April 2025 at the Marine Biotechnology Department of the Ensenada Center for Scientific Research and Higher Education (CICESE; Ensenada, Baja California, Mexico). The study evaluated three dietary treatments: (1) plant-based diet supplemented with two *Bacillus* strains (Plant), (2) fishmeal-based diet supplemented with the same probiotics (Fish), and (3) control diet consisting of fresh giant kelp *M. pyrifera* (Kelp) collected weekly from local coastal waters.

Non-probiotic formulated diet controls were deliberately excluded based on prior experimental evidence and bioethical considerations. Previous trials with similar plant-based formulations lacking *Bacillus* supplementation resulted in 30–40% mortality in different marine organisms [20,33], consistent with the known deleterious effects of unmitigated anti-nutritional factors in high soybean meal diets. The use of 3-year-old abalone—representing substantial biological and economic investment—made exposure to treatments with documented mortality risk ethically unjustifiable under institutional animal welfare guidelines. The experimental design therefore evaluated probiotic-supplemented formulated diets as a complete nutritional strategy against fresh *M. pyrifera* as the commercial reference diet, which constitutes the scientifically and practically relevant comparison for abalone aquaculture development.

A total of 297 *H. rufescens* with a mean (±SD) shell length of 48.51 ± 4.64 mm and body weight of 16.01 ± 5.51 g were obtained from the Aquaculture Department at CICESE. Animals used in this study and all experimental procedures complied with institutional animal welfare guidelines certified by the bioethics committee from CICESE (Ensenada, Baja California, Mexico, ORGA_ACUA_2025_03). Organisms were randomly distributed across nine 200 L circular fiberglass tanks (*n* = 33 per tank) in a factorial design with three dietary treatments and three replicate tanks per treatment. Each tank was equipped with two corrugated 30 × 30 cm plastic refuge plates to provide habitat.

A flow-through seawater system maintained water quality with a 30% daily water exchange rate. Water quality parameters were monitored daily and maintained within optimal ranges for red abalone cultivation [4]. Seawater temperature (17 ± 1 °C) was maintained with ½ HP water chillers (Aqua Logic DS-5, San Diego, CA, USA; controller differential ± 0.55 °C). Dissolved oxygen (>6 mg/L) and pH (8.0–8.2) were measured with a Hatch multimeter (HQ40D, Loveland, CO, USA; DO accuracy: ±0.1 mg L^−1^; pH accuracy: ±0.02). Salinity (33–35‰) was verified using a handheld salinity refractometer with automatic temperature compensation (RHS-10ATC, YHEQUIPMENT, Shenzhen, China; accuracy: ±1‰). Natural photoperiod was maintained (approximately 14:10 light/dark during experimental months) without artificial illumination. Animals were fed the three diets ad libitum, and uneaten feed was removed every two days by siphoning debris from the bottom of each tank before the addition of new food.

### 2.2. Probiotic-Enhanced Feed Formulation and Preparation

#### 2.2.1. Bacillus Probiotic Strains

*Bacillus* Sp1 and Sp3 strains were obtained from a culture collection at the Marine Biotechnology Department of CICESE. They were selected based on their demonstrated capacity to produce carbohydrases, proteases and lipases that act on complex plant polysaccharides, including those found in soybean meal. The phylogenetic identification and growth conditions of *Bacillus* Sp1 and Sp3 strains were previously described by Macías et al. [19] and Mercado et al. [20]. Sp1 and Sp3 were identified as *B. velezensis* and *B. amyloliquefaciens*, respectively; therefore, both strains fall within the Generally Recognized As Safe (GRAS) classification. GRAS is a designation used by the U.S. Food and Drug Administration (FDA), indicating that a substance is considered safe by qualified experts under its intended conditions of use and is exempt from the standard premarket approval required for food and feed additives. For aquafeed applications, GRAS status is practically relevant because it supports the regulatory acceptability of these *Bacillus* species as feed ingredients and reduces barriers to commercial adoption. Strains were cultured at 37 °C and 250 rpm in Schaeffer medium until the T4 sporulation phase was reached; strains were then kept at 4 °C until use.

#### 2.2.2. Feed Composition and Design

The CICESE-prepared feeds (Table 1), which we refer to as plant and fish diets, are non-commercial proprietary recipes, and complete ingredient composition cannot be provided. Both formulated diets were nutritionally equivalent in macro- and micronutrient content. The fish diet had 30% fishmeal as its primary source of protein, with no soybean meal added. In the plant-based diet, only 10% fishmeal was included; soybean meal and other plant meals were used to achieve the same total protein content. Fresh *M. pyrifera* served as a control diet used in commercial aquaculture.

The plant-based diet contained the anti-nutritional factors characteristic of soybean and other plant meals, previously characterized by our group [20]. These include the Kunitz trypsin inhibitor and lectins, which reduce proteolytic digestion and can impair intestinal nutrient absorption; the storage-protein allergens glycinin and β-conglycinin; the α-galactosides raffinose, stachyose, and verbascose, which monogastric organisms cannot hydrolyze in the absence of α-galactosidase; and phytate. These factors were the explicit rationale for the *Bacillus* supplementation: strains Sp1 and Sp3 were selected for their complementary proteases and carbohydrases (including α-galactosidase activity), which degrade these anti-nutritional compounds and improve the bioavailability of plant-derived nutrients.

Both formulated diets maintained equivalent macronutrient profiles: 21% protein, 55% carbohydrates, and 5% lipids to ensure nutritional equivalence and isolate the effects of ingredient source and probiotic enhancement. Pellets were produced and dried at 64 °C for 4 h. Fishmeal was obtained from a local supplier (Procesadora Mar de Ensenada, S. de R.L. de C.V., Ensenada, Baja California, Mexico). Vitamin and mineral premix (Brovel S.A. de C.V., Mexico City, Mexico), soybean meal (Soyarin^®^, FRACA S.A. de C.V., Tepatitlan, Mexico), and wheat flour (El Rosal^®^ Molinera del Valle S.A. de C.V., Mexicali, Mexico) were obtained from national suppliers. Amino acids of principal protein ingredients can be found in [34,35,36].

**Table 1:** Proximal composition of experimental diets and control.

Component	Plant-Based Diet + Probiotics	Fish-Based Diet + Probiotics	Control Fresh *Macrocystis pyrifera*
Macronutrient Composition (%)			
Protein	21.0	21.0	7.67–9.41 ^1^
Carbohydrates	55.0	55.0	47.2–75.3 ^1^
Lipids	5.0	5.0	0.86–0.99 ^1^
NDF	3	3	6.92–7.15 ^1^
DF	5	5	
Gross energy	16.4 kJ g^−1^ (3.92 kcal g^−1^)	16.4 kJ g^−1^ (3.92 kcal g^−1^)	≈10.3–15.6 kJ g^−1^ (mean ~12.9)
Primary Ingredients (%)			
Fishmeal ^2^	10.0	30.0	0
Soybean meal ^3^	10	0	0
Vegetable meals ^4^	74.0	64.0	0
Vitamin and mineral premix ^5^	2.0	2.0	n.a
*Bacillus* strains (Sp1 + Sp3)	2 × 10^6^ CFU/g	2 × 10^6^ CFU/g	0

^1^ Values from Rodríguez-Montesinos and Hernández-Carmona [37] for the Ensenada region where *M. pyrifera* was obtained for this study. Ranges reflect seasonal variations in *M. pyrifera* composition; ^2^ Procesadora Mar de Ensenada, S. de R. L. de C. V.; ^3^ Soyarin^®^; ^4^ De la Rosa^®^; ^5^ Brovel S.A. de C.V.; NDF = Non-Digestible Fiber; DF = Digestible Fiber.

#### 2.2.3. Probiotic Incorporation and Processing

A standardized combination of *Bacillus* strains Sp1 and Sp3 (1:1 ratio) was incorporated into both experimental diets at a concentration of 2 × 10^6^ CFU/g of feed based on previous aquafeed production [13]. Macro- and micronutrient ingredients were thoroughly mixed using a ribbon mixer for 15 min to ensure homogeneous distribution. The probiotic inoculum was then added as a liquid suspension and mixed for an additional 10 min. Pellets were formed using a laboratory-scale pelletizer and subsequently dried at 64 °C for 4 h. The thermal processing conditions were specifically chosen to maintain probiotic viability, as Sp1 and Sp3 strains demonstrate thermostability, surviving temperatures up to 80 °C for extended periods [20]. Post-processing viability analysis confirmed that both *Bacillus* strains maintained concentrations of 1.8–2.2 × 10^6^ CFU/g in dried feeds, as described in Olmos et al. [22].

### 2.3. Growth Performance and Survival Assessment

Biometric assessments were conducted at 0, 90 and 180 days. All organisms were measured for shell length using digital calipers (Mitutoyo 500-196-30, Mitutoyo, Kanagawa, Japan; precision ± 0.1 mm) and body weight using an analytical balance (precision ± 0.01 g). Individual growth metrics were measured as total growth (mm, g), daily (DGR; µm/day, g/day) and monthly (MGR; mm/month, g/month) growth rates. Mortalities were recorded daily, with dead animals removed and not replaced, and summarized at biometric sampling points.

### 2.4. Postprandial Metabolism Assessment

#### 2.4.1. Respirometry Experimental Design

Following the 180-day growth trials, postprandial metabolic responses were assessed through intermittent-flow respirometry. Twenty abalone per dietary treatment were used (Plant: 49.00 ± 3.83 g, mean ± SD; Fish: 43.94 ± 5.91 g; Kelp: 53.24 ± 3.44 g). Size differences among treatment groups at this point were a consequence of 180 days of differential dietary-driven growth and therefore reflect the biological reality of the experimental treatments. To account for these differences, all metabolic rates were normalized to soft tissue mass using the allometric regression equation from Searle et al. [31]: Soft tissue mass (g) = 0.557 × Total weight (g) (R^2^ = 0.99). It also removes the metabolically inert shell mass, which represents approximately 44.3% of total abalone weight. All metabolic rates are expressed per gram of soft tissue.

The system consisted of 10 acrylic respiratory chambers (500 mL volume each), equipped with valves to control filtered seawater flow. Each chamber contained a mini optical oxygen sensor (Loligo Systems, Viborg, Denmark) connected to a fiber optic transmitter (OXY-10 mini, PreSens, Regensburg, Germany) controlled by OXY-10 mini software version 3.33FB.

Animals were fed their respective diets 24 h prior to measurements. Chambers were filled with filtered seawater (17 ± 1 °C), and one abalone was placed in each chamber (*n* = 10). Water flow was closed and oxygen consumption was measured every 5 min for 6 continuous hours or until oxygen concentration reached 75% saturation to avoid stress. When any chamber reached 75% saturation, water flow was opened and maintained for 10 min until 100% saturation was restored, after which measurements resumed. This procedure was performed in duplicate for each diet to achieve *n* = 20 per measurement time point. Measurements were conducted at eight time points: hours 25, 26, 27, 28, 29, and 30 post-feeding (postprandial period), and hours 72 and 73 post-feeding (post-absorptive period).

#### 2.4.2. Oxygen Consumption Rate and Relative Specific Dynamic Action (SDA)

Postprandial and postabsorptive oxygen consumption rates (MO_2_) were calculated from the decline in dissolved oxygen concentration during the closed phase of the respirometry cycle, following standard mass balance approaches used in aquatic respirometry and intermittent-flow respirometry [38]. MO_2_ was expressed as mg O_2_/g soft tissue/h and calculated using Equation (1):

(1) $${MO}_{2}=\frac{{([O}_{2}{]}_{initial}-{[O}_{2}{]}_{final})\times V}{\left(W\times T\right)}$$

where [O_2_]_initial_ − [O_2_]_final_ was the difference in dissolved oxygen concentration (mg O_2_/L); V was the chamber volume in liters minus the volume displaced by the abalone; W was the g of soft tissue; and T was the measurement time in hours.

Specific Dynamic Action (SDA) describes the postprandial increase in metabolic rate associated with ingestion, digestion, absorption, and assimilation of a meal [26,39]. In this study, we estimated a relative SDA index as the percentage increase in mean postprandial MO_2_ relative to the post-absorptive baseline, rather than as the total integrated postprandial oxygen cost. Relative SDA was calculated using Equation (2) and expressed as %:

(2) $$SDA\left(\%\right)=\frac{\left(\mathrm{Mean}\;\mathrm{postprandial}\;{\mathrm{MO}}_{2}-\mathrm{Mean}\;\mathrm{postabsorptive}\;{\mathrm{MO}}_{2}\right)}{\left(\mathrm{Mean}\;\mathrm{post}-\mathrm{absorptive}\;{\mathrm{MO}}_{2}\right)}\times 100$$

where mean postprandial MO_2_ represents average oxygen consumption during hours 25–30 post-feeding, and mean post-absorptive MO_2_ represents average oxygen consumption during hours 72–73 post-feeding.

#### 2.4.3. Ammonia Excretion Rate

Simultaneously with oxygen consumption measurements, 10 mL water samples (*n* = 5 per replicate × 2 replicates = 10 total) were collected before closing and after reopening water flow to respiratory chambers to determine initial and final ammonia concentrations. All samples were analyzed individually using the Nessler method with a low-range ammonia colorimeter (0.00 to 3.00 ± 0.05 ppm NH_3_-N) (Hanna, HI700 Checker HC, Woonsocket, RI, USA) and two high-quality reagents (Hanna, HI93700A, HI93700B-0, Woonsocket, RI, USA). For seawater samples, eight drops of each reagent were used as recommended by the manufacturer. Colorimeter readings were retained as NH_3_-N.

Ammonia excretion rate (AER) was calculated from the change in ammonia-nitrogen concentration over time, corrected by chamber volume and soft tissue mass, following standard approaches used in aquatic physiological energetics [40]. AER was expressed as mg NH_3_-N/g soft tissue/h and calculated using Equation (3):

(3) $$AER=\frac{\left([{\mathrm{NH}}_{3}-N{]}_{final}-[{\mathrm{NH}}_{3}-N{]}_{initial}\right)\times V}{\left(W\times T\right)}$$

where [NH_3_-N]_final_ − [NH_3_-N]_initial_ was the difference in ammonia concentration (mg NH_3_-N/L); V was the chamber volume in liters minus the volume displaced by the abalone; W was the grams of soft tissue; and T was the measurement time in hours.

#### 2.4.4. O:N Ratio

The oxygen:nitrogen (O:N) ratio was calculated as the ratio between oxygen consumed and ammonia-nitrogen excreted, both expressed in equivalents [41]. The O:N ratio was calculated using Equation (4):

(4) $$O:N=\frac{\left({MO}_{2}/16\right)}{\left(AER/14\right)}$$

where MO_2_ is the oxygen consumption rate expressed as mg O_2_/g soft tissue/h, and AER is the ammonia excretion rate expressed as mg NH_3_-N/g soft tissue/h. Divisor 16 corresponds to the mass of oxygen, while divisor 14 corresponds to the mass of nitrogen. The resulting O:N ratio is dimensionless and was used as an indicator of the relative contribution of nitrogenous and non-nitrogenous substrates to aerobic metabolism [41,42,43].

### 2.5. Statistical Analysis

Analyses were performed in R 4.0.5 (packages tidyverse, nlme, emmeans, and car; figures in ggplot2). Data are presented as mean ± standard error unless otherwise noted, and significance was set at α = 0.05. Normality and homogeneity of variance were verified with Shapiro–Wilk and Levene’s tests. Individual abalone were the unit of observation; because animals were housed by treatment in three tanks per diet, the tank was treated as the experimental unit and modeled as a random effect to avoid pseudoreplication.

Growth trajectories (shell length and weight at days 0, 90, and 180) were analyzed with linear mixed-effects models (LME) of the form *Response*~*Diet* × *Time* + (1|*Tank*), with diet, time, and their interaction as fixed effects and tank as a random effect. The significance of fixed effects was tested by Type III analysis of variance (ANOVA). Final-day (180) shell length, weight, and daily growth rate were compared among diets by one-way ANOVA.

Metabolic rates—oxygen consumption, the relative SDA index, and ammonia excretion—were analyzed with LME fitted by restricted maximum likelihood, with diet, period (postprandial, post-absorptive), and their interaction as fixed effects, and a random effect for the repeated measurements taken over time.

For all models, when a significant interaction was detected, pairwise comparisons—among diets within each period and between periods within each diet—were made on estimated marginal means with Tukey’s honestly significant difference (HSD) adjustment for multiple comparisons.

The O:N ratio was computed as the atomic ratio of mean oxygen consumption to mean ammonia-nitrogen excretion within each diet and period (ratio-of-means) and summarized with 95% confidence intervals from 10,000 bootstrap resamples; within-diet temporal contrasts were assessed by the same bootstrap procedure. Because oxygen and ammonia were quantified on separate subsamples, O:N was treated as a derived descriptive index, and inference on substrate-use differences was based primarily on the mixed-effects models of oxygen consumption and ammonia excretion.

## 3. Results

### 3.1. Growth Performance and Survival

Total growth during the 180-day period was 12.41 ± 0.69 mm for the plant diet, 8.97 ± 0.78 mm for the fish diet, and 18.06 ± 0.67 mm for the fresh kelp control. This resulted in kelp treatment producing the highest growth rate (3.01 ± 0.11 mm/month; 100.35 ± 3.73 μm/day), followed by plant diet (2.07 ± 0.11 mm/month; 68.93 ± 3.81 μm/day), while fish diet resulted in the lowest growth performance (1.49 ± 0.13 mm/month; 49.82 ± 4.31 μm/day). Cumulative mortality over 180 days was low in all treatments: 3 of 99 animals in both the kelp and plant treatments (96.97% survival) and 6 of 99 in the fish treatment (93.94%) (Table 2). Survival did not differ significantly among treatments (χ^2^ = 1.56, df = 2, *p* = 0.46). All kelp and fish deaths occurred during days 90–180, whereas plant-treatment mortality was distributed across both intervals (2 deaths during days 0–90, 1 during days 90–180). Final tank-level sample sizes ranged from 29 to 33 individuals; this minor imbalance was accommodated by the inclusion of the tank as a random effect in the linear mixed-effects models.

**Figure 1:**
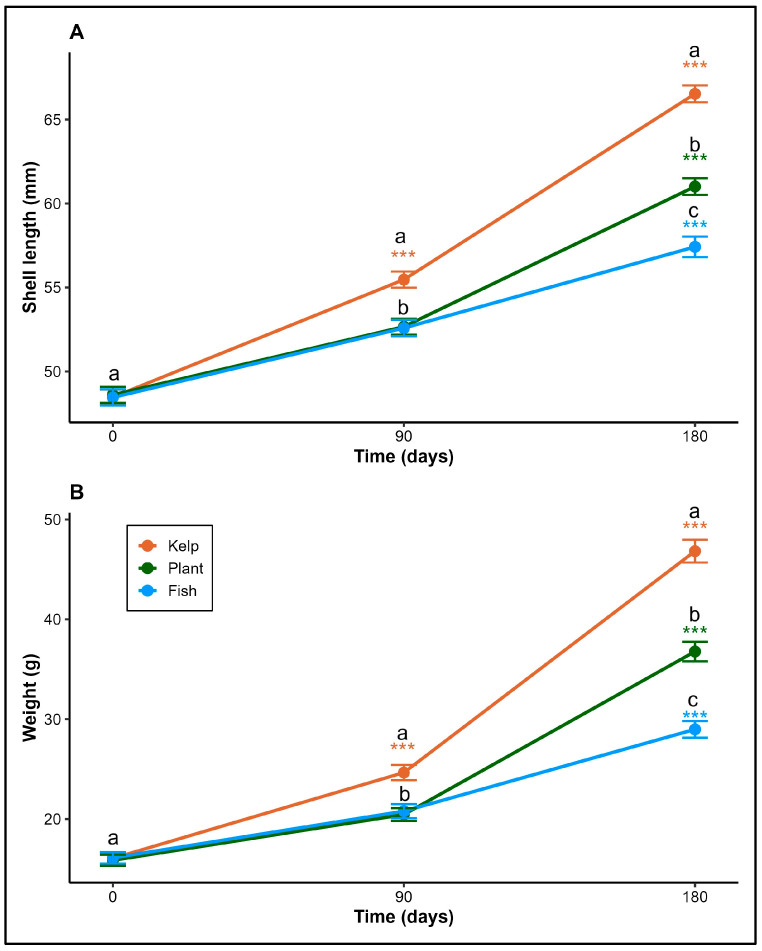
*Haliotis rufescens* growth in shell length (mm, (**A**)) and weight (g, (**B**)) during the 180-day experiment. Plant + *Bacillus* diet in green, fish + *Bacillus* diet in blue, and control fresh giant kelp *Macrocystis pyrifera* in brown. Values are indicated as mean ± SE. One-way analysis of variance with letters indicating statistical differences; *** = *p* < 0.001.

On day 90, diet treatments had a significant effect on abalone size (one-way ANOVA, F = 11.69, df = 2, *p* < 0.001) and weight (one-way ANOVA, F = 10.99, df = 2, *p* < 0.001). Tukey HSD tests indicated that kelp treatment differed significantly from plant and fish treatments for both size and weight parameters. At day 180, diet also significantly influenced abalone size (one-way ANOVA, F = 73.11, df = 2, *p* < 0.001) and weight (one-way ANOVA, F = 80.81, df = 2, *p* < 0.001). Tukey HSD tests indicated significant differences among all treatment groups for both size and weight parameters (Figure 1A,B).

### 3.2. Oxygen Consumption

**Figure 2:**
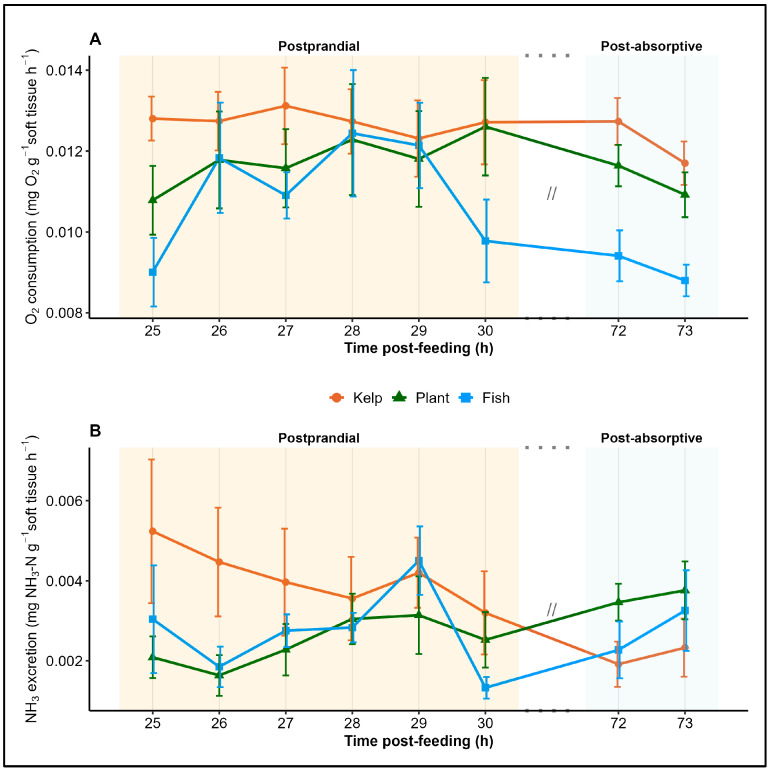
Oxygen consumption rates (**A**) and ammonia excretion (**B**) in *Haliotis rufescens* fed control fresh giant kelp *Macrocystis pyrifera* (brown), plant + *Bacillus* (green), or fish + *Bacillus* (blue) diets over 73 h. Light orange color indicates the postprandial (25–30 h) and light blue post-absorptive (72–73 h) periods. Oxygen consumption differed significantly among diets (Linear mixed-effects, F_2,468_ = 9.69, *p* < 0.001); ammonia excretion showed a significant diet × period interaction (F_2,228_ = 4.97, *p* = 0.008). Values are mean ± SE.

Oxygen consumption rates differed significantly among dietary treatments (LME, F_2,468_ = 9.69, *p* < 0.001), with kelp-fed abalone exhibiting the highest metabolic rates during both postprandial (0.0127 ± 0.00034 mg O_2_/g soft tissue/h) and post-absorptive periods (0.0122 ± 0.00040 mg O_2_/g soft tissue/h). Plant-fed abalone showed intermediate rates (postprandial: 0.0118 ± 0.00046; post-absorptive: 0.0113 ± 0.00038 mg O_2_/g soft tissue/h), while fish diet-fed abalone exhibited lower oxygen consumption (postprandial: 0.0110 ± 0.00046; post-absorptive: 0.0091 ± 0.00037 mg O_2_/g soft tissue/h; Figure 2A).

Pairwise comparisons revealed that kelp-fed abalone consumed significantly more oxygen than fish-fed abalone during both the postprandial (*p* = 0.005) and post-absorptive periods (*p* = 0.003). Differences between kelp and plant treatments were not significant (*p* >0.20 for both periods) (Table 3). Interestingly, fish treatment was less stable than both plant and kelp treatments, showing high consumption peaks followed by drops in oxygen consumption during the postprandial period (Figure 2A), which resulted in different SDA values.

### 3.3. Postprandial Metabolic Response

The SDA, representing the metabolic cost of feed processing, varied among treatments (Table 3). Fish-fed abalone exhibited an SDA of 21.0%, indicating substantial energetic costs associated with diet digestion and nutrient absorption. In contrast, both kelp-fed (4.3%) and plant-fed abalone (4.7%) demonstrated minimal postprandial metabolic increases, suggesting efficient digestion and nutrient assimilation (Table 3). The five-fold higher SDA in fish-fed abalone compared to kelp and plant treatments reflects reduced feed conversion efficiency and elevated metabolic costs associated with fishmeal-based diet processing.

### 3.4. Ammonia Excretion

Ammonia excretion patterns revealed a significant diet × period interaction (F_2,228_ = 4.97, *p* = 0.008), indicating distinct temporal metabolic strategies among treatments. During the postprandial period, kelp-fed abalone exhibited the highest ammonia excretion (0.00410 ± 0.00051 mg NH_3_/g soft tissue/h), significantly exceeding both plant-fed (0.00245 ± 0.00027 mg NH_3_/g soft tissue/h; *p* = 0.004) and fish-fed abalone (0.00272 ± 0.00031 mg NH_3_/g soft tissue/h; *p* = 0.018). Plant and fish treatments showed similar postprandial excretion rates (*p* = 0.859; Table 3; Figure 2B).

During the post-absorptive period, temporal patterns diverged markedly among diets. Kelp-fed abalone showed a significant 48.5% reduction in ammonia excretion to 0.00212 ± 0.00045 mg NH_3_/g soft tissue/h (*p* = 0.036). In contrast, plant-fed abalone exhibited a 47.3% increase to 0.00361 ± 0.00042 mg NH_3_/g soft tissue/h. Though this increase was non-significant (*p* = 0.165), it has important metabolic perspectives. These variations between kelp and plant treatments potentially indicate differences in metabolic strategies and utilization efficiencies of energy sources obtained from protein and carbohydrate degradation. Fish-fed abalone showed the same ammonia excretion across both periods (*p* = 0.953) with a similar postprandial pattern to that of plant-fed abalone (Table 3; Figure 2B). However, fish-fed abalone showed a less stable pattern, with sudden peaks and drops of shorter duration in ammonia excretion, mostly at 26 and 30 h post-feeding (Figure 2B). This contrasted with more stable excretion of the kelp and plant treatments and mirrored patterns seen in oxygen consumption (Figure 2A). Together, these differences produced a distinct O:N ratio for the fish treatment.

### 3.5. O:N Ratio and Metabolic Substrate Utilization

**Figure 3:**
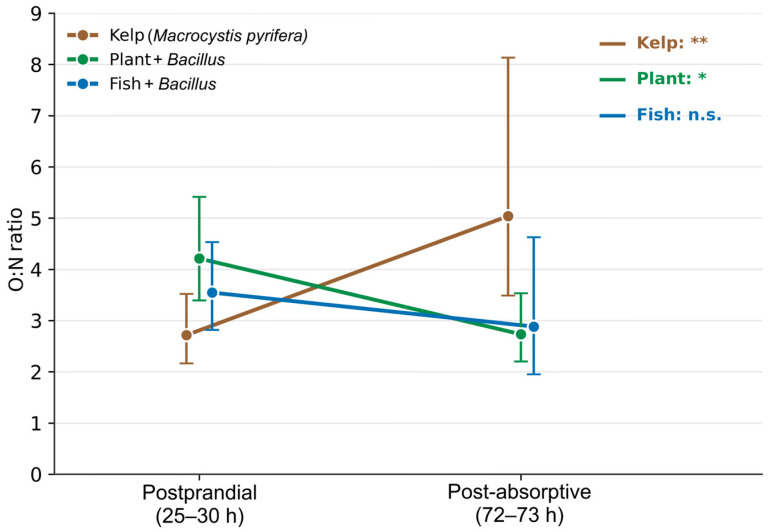
Oxygen-to-Nitrogen (O:N) ratio during postprandial (25–30 h) and post-absorptive (72–73 h) periods in abalone fed kelp (brown), plant + *Bacillus* (green), or fish + *Bacillus* (blue). Values are means, and error bars are 95% bootstrap confidence intervals with 10,000 resamples. Significance is within-diet postprandial versus post-absorptive contrast (** = *p* < 0.01; * = *p* < 0.05; n.s. = non-significant).

The O:N ratio exhibited a significant diet × period interaction (F_2,228_ = 3.901, *p* = 0.022), revealing diet-specific shifts in metabolic substrate preferences over time. Kelp-fed abalone demonstrated a unique metabolic pattern, significantly increasing their O:N ratio from 2.72 (2.17–3.52) during the postprandial period to 5.04 (3.49–8.13) in the post-absorptive phase (*p* = 0.005), suggesting a metabolic shift away from protein catabolism toward greater reliance on carbohydrate oxidation (Table 3; Figure 3).

Conversely, plant-fed abalone showed an opposite trend, with the O:N ratio decreasing from 4.21 (3.38–5.38) during the postprandial period to 2.73 (2.20–3.51) in the post-absorptive phase (*p* = 0.011). Fish-fed abalone showed an intermediate pattern, with an O:N ratio of 3.55 (2.82–4.53) during the postprandial period and 2.88 (1.96–4.67) during the post-absorptive phase (*p* = 0.447). Compared with the plant treatment, the fish-fed group showed more pronounced short-term fluctuations in O:N ratio during the postprandial period (Figure 3).

## 4. Discussion

This study evaluates growth performance and postprandial metabolism in sub-adult *H. rufescens* fed three distinct dietary regimes. Our findings indicate that a plant-based diet supplemented with *Bacillus* probiotics elicits a metabolic and growth response comparable to natural fresh *M. pyrifera*, while supporting enhanced reproductive development. In contrast, the fishmeal-based diet, despite identical macronutrient and probiotic profiles, resulted in significantly lower growth and metabolic inefficiency. These results underscore the importance of dietary ingredient sources complemented with probiotics, not just composition, and the role of metabolic profiling in developing sustainable aquafeeds.

### 4.1. Growth Performance and Energetic Trade-Offs

The divergent growth trajectories observed reflect fundamental differences in energy allocation strategies driven by dietary composition (Figure 1). The superior somatic growth in kelp-fed abalone (3.01 mm/month; 100 μm/day) aligns with the evolutionary diet for *H. rufescens*, providing nutrients in a highly bioavailable form that prioritizes tissue accretion [4]. Notably, this growth rate exceeds the average reported in the literature for sub-adult red abalone fed fresh *M. pyrifera* in laboratory settings (0.45–1.5 mm/month; 15–50 μm/day) [4,44,45], land-based farm (1.8 mm/month; 60 μm/day; R. Flores, Abulones Cultivados Eréndira Baja California Mexico, pers. comm.), or mariculture (2.64 mm/month; 88 μm/day) [3]. However, reproductive development was minimal in the kelp diet, suggesting preferential energy allocation toward somatic growth.

The plant-based diet produced commercially viable growth (2.07 mm/month; 69 μm/day), coupled with enhanced reproductive development, indicating a strategic partitioning of energy toward somatic growth and reproductive investment. This trade-off between somatic and reproductive growth is a well-documented physiological response in abalone [46,47,48], suggesting that our plant-based formulation provides a nutritional matrix conducive to broodstock conditioning while maintaining adequate somatic growth [22]. The growth obtained with the plant-based diet exceeded that demonstrated by Kemp et al. [49], who obtained 39 μm/day in *H. rufescens* fed a South African commercial abalone feed (Marifeed, Pty Ltd., Hermanus, Western Cape, South Africa) containing fishmeal and soybean meal as main sources. Additionally, it is important to note that *H. rufescens* used in their study were juveniles (20–26 mm; 1–3 g), smaller than the sub-adults used in our study (48–66 mm; 16–46 g), which diverted energy into reproduction, not only somatic growth.

Conversely, the fishmeal-based diet yielded the poorest growth (1.49 mm/month; 50 μm/day), a finding that initially appears paradoxical given its high-quality protein content, amino acid profile, and high survival rate (94%). Our results are in agreement with Riddin [50] and Wu et al. [51], who documented that *H. midae* fed a diet of fishmeal and soybean meal combination grew better on diets combining fishmeal and soybean meal compared with fishmeal as the sole protein source. In that sense, poor performance cannot be attributed to palatability issues or compromised animal health but rather could reflect metabolic inefficiencies in nutrient utilization—as revealed by oxygen consumption, SDA and ammonia excretion measurements. This outcome aligns with previous observations that excessive dietary protein from animal sources often leads to catabolism for energy rather than anabolism, while simultaneously inducing nitrogen homeostasis imbalance [10,52].

### 4.2. Metabolic Efficiency Related to Oxygen Consumption and SDA

Postprandial oxygen consumption rates and SDA provide mechanistic explanations for the observed growth disparities among dietary treatments. Oxygen consumption postabsorptive rate differed significantly among diets, with kelp-fed abalone exhibiting the highest rates, followed by plant-fed and fish-fed abalone. These patterns reflect distinct strategies of energy management tied to dietary composition and temporal dynamics of nutrient processing.

Kelp-fed abalone displayed elevated oxygen consumption during both postprandial and post-absorptive periods, consistent with simultaneous processing of carbohydrates and proteins of macroalgal tissue [27,53,54]. This high metabolic demand likely reflects the energetic cost of processing a large nutrient influx. However, this cost appears to be offset by the high bioavailability of kelp-derived compounds and by the evolutionary adaptation of abalone’s digestive system. In particular, endogenous polysaccharide-degrading enzymes such as amylase, cellulase, laminarinase, and alginase can efficiently process kelp [10,27,53,55].

Despite these elevated oxygen consumption post-absorptive rates, kelp-fed abalone exhibited low SDA (4.3%), suggesting minimal additional metabolic cost associated with postprandial digestion. This low SDA indicates that *M. pyrifera* is well-matched to abalone’s digestive physiology, allowing efficient extraction and assimilation of nutrients with minimal energetic costs [10,55]. Importantly, this metabolic efficiency does not translate to favorable feed conversion efficiency. The requirement of 17–20 kg of fresh kelp to produce 1 kg of abalone biomass [52] indicates that much of the ingested material—particularly complex carbohydrates—is excreted without being incorporated into biomass or energy reserves.

This paradox can be explained by abalone’s metabolic strategy: the animal preferentially digests proteins and simple carbohydrates while excreting substantial quantities of indigestible polysaccharides [27]. The result is metabolically efficient processing of a limited nutrient fraction, rather than comprehensive nutrient utilization across the entire ingested biomass. Consequently, while kelp supports high growth rates, the energetic and logistical costs of collecting, transporting, and providing large quantities of fresh kelp present constraints for commercial aquaculture operations. This is critically important considering the loss of > 90% of *M. pyrifera* populations in Baja California in the last decade [6].

Plant-fed abalone exhibited intermediate oxygen consumption postabsorptive rates and maintained a similarly low SDA (4.7%) to kelp-fed abalone, suggesting efficient energy management. This metabolic efficiency could be related to the enzymatic activities of *Bacillus* strains Sp1 and Sp3, which produce carbohydrases and proteases capable of degrading complex plant-derived substrates [18,19]—including soybean oligosaccharides, protease inhibitors, and starch from other plant meals—that would otherwise be refractory to abalone’s endogenous enzymes [18,21,56,57]. Additionally, *Bacillus* species generate short-chain fatty acids (SCFAs) such as acetate, propionate, and butyrate through fermentation of complex carbohydrates [58]. These SCFAs can lower intestinal pH, enhancing protein digestion, while simultaneously inhibiting pathogen colonization [59]. The combined effects of exogenous enzymatic supplementation and beneficial microbial metabolite production likely explain why the plant-based diet supplemented with probiotics achieved SDA values comparable to *M. pyrifera* despite originating from terrestrial sources. This finding is consistent with previous studies demonstrating that probiotic supplementation in abalone enhances growth performance, stabilizes gut microbiome composition, and upregulates digestive enzyme activities [57,60,61].

Conversely, fish-fed abalone exhibited elevated SDA (21.0%), a five-fold increase relative to both kelp and plant treatments. This energetic cost represents metabolic inefficiency wherein approximately 17% more of the assimilated energy could be diverted to digestion-related processes rather than being available for growth. Elevated SDA can result from multiple interrelated factors, including suboptimal nutrient composition, excessive protein deamination, elevated nitrogenous waste excretion, and osmotic or pH disturbances associated with rapid amino acid absorption [26,27].

In the present study, the high SDA of the fishmeal diet suggests a mismatch between the diet’s compositional profile and the abalone’s herbivorous digestive physiology. Fishmeal protein, while of high quality for carnivorous species, delivers concentrated amino acids that appear to exceed the protein synthesis capacity of abalone [62], potentially resulting in catabolism of excess amino acids for energy rather than anabolic incorporation into tissue. Furthermore, fishmeal contains approximately three times higher concentrations of purines compared to plant-based protein sources [63]. Purines must be catabolized and excreted as uric acid or ammonia, imposing additional metabolic costs without contributing to growth [64]. Consequently, elevated purine content of fishmeal exacerbates nitrogen homeostasis stress and increases the energetic cost of waste nitrogen elimination.

Additionally, the fishmeal diet—despite the same concentration of *Bacillus* probiotics—potentially offered limited substrates for carbohydrase activity due to its lower overall carbohydrate complexity, while simultaneously imposing nitrogen homeostasis stress on both the host and probiotic organisms due to excessive amino acid and purine content [13]. In that sense, the elevated SDA observed in fish-fed abalone could reflect a systemic failure in diet–host compatibility, wherein the energetic cost of nutrient processing undermines the potential benefits of high-quality protein. These findings underscore ingredients quality must be evaluated not only by nutrient composition but also by physiological compatibility with the target organism’s digestive and metabolic capacities.

### 4.3. Nitrogen Metabolism and Substrate Use

Ammonia excretion patterns and O:N ratio provide useful, although indirect, information on diet-associated differences in metabolic substrate use in *H. rufescens*. The O:N ratio is commonly interpreted as an index of the relative contribution of nitrogenous versus non-nitrogenous substrates to aerobic metabolism [43]. In general, a low O:N ratio (<16) indicates predominant protein or amino acid catabolism, intermediate values suggest mixed use of protein and lipid substrates, and high values reflect greater reliance on non-protein substrates such as lipids and/or carbohydrates [43,65,66]. Abalone are known to utilize protein and carbohydrate catabolism to meet energetic demand, with minimal contribution from lipid oxidation [10,27,30,53,54]. While O:N ratios below 16 are typically interpreted as indicative of protein catabolism, the values observed (3.80–11.03) could reflect the natural metabolic substrate preferences of herbivorous gastropods consuming low-lipid diets. These ratios do not indicate metabolic limitation or nutritional deficiency. Rather, they reflect the evolved reliance of abalone on protein and carbohydrate oxidation as primary energy sources. This is consistent with their natural diet of macroalgae, which is low in lipids, rich in structural and storage carbohydrates, and moderate in protein [27,30,32,67].

**Figure 4:**
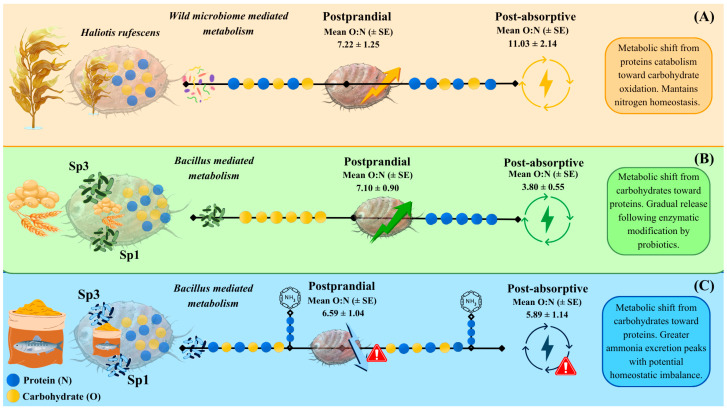
Conceptual model of metabolic routing and energy allocation in sub-adult red abalone (*Haliotis rufescens*) across three diets. Blue circles denote protein (nitrogen source); yellow circles denote carbohydrate (oxygen demand). (**A**) Wild microbiome-mediated metabolism (yellow arrow indicates highest somatic growth). (**B**) Plant-based + *Bacillus* diet: a hypothesized model of probiotic-mediated sequential nutrient release, in which strain Sp3 is proposed to degrade carbohydrates first and strain Sp1 to release plant proteins more gradually; this proposed controlled release could limit nitrogen toxicity and support both somatic and reproductive growth (green arrow indicates highest gonadal maturation and commercially viable somatic growth). (**C**) Fishmeal-based + *Bacillus* diet: metabolic dysfunction (red warning icons) affecting growth (blue arrow indicates lowest somatic growth). Panels depict a conceptual hypothesis consistent with the observed metabolic patterns.

Kelp-fed abalone at the beginning exhibited the highest postprandial ammonia excretion, which decreased by 48.5% to post-absorptive levels. This pattern suggests rapid digestion and absorption of kelp protein following ingestion, with surplus nitrogenous compounds being promptly deaminated and excreted to prevent accumulation and maintain nitrogen homeostasis [30,32]. The corresponding increase in O:N ratio from 2.72 (2.17–3.52) during the postprandial period to 5.04 (3.49–8.13) in the post-absorptive phase (*p* = 0.005) suggests a metabolic shift away from protein catabolism toward greater reliance on carbohydrate oxidation once protein digestion is largely complete (Figure 4). This temporal metabolic flexibility indicates efficient nitrogen management: kelp-fed abalone could process and assimilate dietary protein during the postprandial phase, retain nitrogen for anabolic processes, and then transition to carbohydrate metabolism during the post-absorptive period. This strategy minimizes prolonged exposure to elevated nitrogenous waste while maintaining steady energy availability.

Plant-fed abalone displayed an opposite temporal pattern in nitrogen metabolism. Ammonia excretion during the postprandial period was significantly lower than in kelp-fed abalone, followed by a 47.3% increase during the post-absorptive phase. Correspondingly, the O:N ratio decreased from 4.21 (3.38–5.38) during the postprandial period to 2.73 (2.20–3.51) in the post-absorptive phase. Such a low post-absorptive value is consistent with strong reliance on protein or amino acid catabolism [43]. These results suggest a potential sequential nutrient-release pattern. Carbohydrates would be degraded and absorbed first during the initial postprandial period. Plant proteins—encapsulated within complex polysaccharide matrices and protected by protease inhibitors—would then be released more gradually, once probiotic carbohydrases and proteases have modified these structures (Figure 4). This hypothesis is supported by the known characteristics of the *Bacillus* strains employed [13,18,19,20]. Strain Sp3 exhibits high carbohydrase activity and rapidly degrades complex carbohydrates, while strain Sp1 produces robust proteolytic enzymes but is subject to catabolite repression in the presence of simple sugars [20]. In that sense, during the early postprandial period, Sp3 potentially degrades soy oligosaccharides and other plant-derived carbohydrates, generating simple sugars that are absorbed by the host while simultaneously suppressing Sp1 protease production via catabolite repression. Once simple sugars are depleted, catabolite repression is relieved, potentially allowing Sp1 to produce proteases that degrade plant proteins—now accessible following carbohydrase-mediated breakdown of protective polysaccharide matrices and protease inhibitors. This sequential liberation and absorption of carbohydrates followed by proteins could explain the delayed ammonia excretion peak and sustained protein catabolism observed in plant-fed abalone during the post-absorptive period.

Critically, this delayed protein digestion does not impair growth performance; rather, it likely contributes to sustained amino acid availability over extended periods, supporting continuous protein synthesis for both somatic and reproductive tissue development. Furthermore, the substantial reproductive investment observed in plant-fed abalone suggests that a portion of dietary nitrogen was preferentially allocated to gonadal development rather than exclusively to somatic growth, potentially explaining the lower somatic growth rate relative to kelp-fed abalone while still achieving commercially viable growth performance.

The fishmeal diet had a similar behavior to the plant diet during postprandial and post-absorptive periods, indicating animals fed *Bacillus* diets preferentially utilize carbohydrates at the beginning and proteins at the end of the same period. However, with a fish diet, the rising of ammonia excretion is sudden and of major proportion, yet of shorter duration. This behavior suggests that excess amino acids or their metabolites at a given time may be inducing homeostatic imbalance in the animal or in *Bacillus*, preventing the correct functioning of the metabolism and the consequent decrease in growth (Figure 4). The drastic decrease in oxygen consumption in the fishmeal diet at the same time as the increase in ammonia excretion supports the hypothesis of metabolic imbalance in animals on this treatment. In addition to amino acids, poor nitrogen management in fishmeal-fed abalones and high concentration of purines in fishmeal—which are three times more abundant than in soybean meal— also require deamination and excretion as uric acid or ammonia, potentially imposing additional metabolic costs providing no growth benefit [52,53,63]. In that sense, when dietary protein—particularly from fish sources—exceeds the anabolic capacity, amino acid excess seems to affect the metabolic capacity, both from abalone and probiotics [13]. Our data suggest that the fishmeal diet triggered this inefficient behavior, resulting in wasteful nitrogen excretion and suppressed growth related to nitrogen homeostasis imbalance [13]. This metabolic inefficiency may therefore arise from several factors: (1) the rapid influx of amino acids from highly digestible fishmeal protein exceeds abalone’s anabolic capacity, forcing catabolism of the surplus; (2) the high purine content imposes an additional metabolic burden; and (3) the resulting disruption of nitrogen homeostasis creates systemic metabolic stress that may suppress anabolic pathways while promoting catabolic ones. However, because enzyme activity, amino acid turnover, and nitrogen retention were not directly assessed, this explanation should be considered a plausible interpretation and explored in further studies.

The molecular basis for this metabolic dysfunction in fish-fed abalone likely involves dysregulation of the mechanistic target of rapamycin (mTOR) signaling pathway—the central cellular regulator of amino acid-stimulated protein synthesis. In Pacific abalone (*H. discus hannai*), mTOR signaling has been shown to be highly sensitive to both the concentration and temporal kinetics of dietary amino acid delivery [15,16,24]. Diets with high animal protein concentration and excessive amino acids can suppress the mTOR signaling cascade, downregulating key anabolic genes such as *Akt* and *eIF4e*, thereby inhibiting ribosomal protein synthesis and muscle tissue deposition [15,16]. Conversely, plant-based diets supplemented with probiotics—which enhance protein digestibility in situ while moderating the rate of amino acid release—could effectively activate mTOR signaling, promoting efficient protein synthesis [16]. In that sense, a fishmeal-based diet with excessive nitrogen supply and compounded by high purine content appears to have overwhelmed rather than optimally stimulated abalone’s mTOR-regulated anabolic machinery. This amino acid oversupply likely triggered a metabolic shift from anabolism to catabolism, wherein excess amino acids were oxidized for energy—generating the observed high SDA—while nitrogenous waste was continuously excreted as ammonia. Under these conditions, the mTOR pathway would be actively suppressed in response to the dual metabolic burden of nitrogen excess and purine catabolism, creating a state of anabolic resistance that prevents efficient conversion of dietary protein into somatic growth. In that sense, these patterns support that diet source affected postprandial metabolic routing, but the specific biochemical mechanisms underlying these responses require further confirmation through measurements of digestive enzyme activity, energy reserves, amino acid metabolism, and tissue nitrogen retention.

### 4.4. Implications for Aquafeed Development

The findings of this study provide insights into the development of economically viable and ecologically sustainable abalone aquafeeds. First, terrestrial plant-based protein sources, particularly when combined with targeted probiotic supplementation, could represent a promising alternative to fresh macroalgae and fishmeal across growth performance, feed conversion efficiency, reproductive development, and operational feasibility. The plant-based diet supported commercially acceptable growth rates (2.07 mm/month) while achieving a more favorable feed conversion ratio than fresh kelp—on the order of 2 kg of dry formulated feed per kg of biomass gain, compared with the 17–20 kg of fresh kelp per kg [8, R. Flores, Abulones Cultivados Eréndira, Baja California, Mexico, pers. comm.]. Simultaneously, this formulation promoted substantial reproductive maturation [22], serving dual-purpose applications in both market-size grow-out operations and broodstock conditioning programs. The logistical and economic advantages of formulated feeds—including year-round availability, standardized nutrient composition, reduced labor costs for collection and transport, and extended shelf life—further support the potential commercial viability of plant-based aquafeeds relative to wild-harvested kelp [8].

Second, protein requirements for abalone typically range from 20 to 50% of total nutrient intake [52,68,69]. This study suggests that protein quantity alone is insufficient to optimize abalone nutrition; rather, protein source and the temporal dynamics of amino acid delivery are equally important. Our results suggest that a moderate protein inclusion level (21%) from predominantly plant-based sources, when supplemented with probiotics that potentially enhance digestibility and modulate nutrient release kinetics, is adequate—and could be superior—to higher-protein fishmeal content formulations for herbivorous gastropods. These findings challenge conventional aquafeed paradigms that prioritize high fishmeal inclusion.

Finally, the efficacy of *Bacillus* probiotic supplementation in enhancing plant-based diet performance highlights the potential of functional feed additives to overcome traditional limitations of terrestrial plant ingredients, including ANFs, suboptimal amino acid profiles, and complex structural polysaccharides. Sp1 and Sp3 strains employed in this study were selected based on their complementary enzymatic profiles and demonstrated capacity to improve growth performance, digestive efficiency, immune function, and microbiome stability in multiple aquaculture species [13,19,20,22]. However, the mechanisms by which these probiotics enhance reproductive development, modulate amino acid absorption kinetics, and interact with host mTOR signaling pathways remain incompletely understood and warrant further investigation through transcriptomic, proteomic, and metabolomic approaches.

## 5. Conclusions

This study indicates that dietary ingredient composition—not simply macronutrient ratios—determines metabolic efficiency, energy management strategies, and production outcomes in *H. rufescens* aquaculture. Fresh kelp *M. pyrifera* obtained the highest growth rates (3.01 mm/month); however, logistical constraints associated with wild harvest limit the scalability and economic viability of kelp as the sole feed source for commercial abalone production.

In contrast, the plant-based diet supplemented with *Bacillus* probiotics emerges as a potential sustainable alternative, achieving commercially viable growth rates (2.07 mm/month) and higher reproductive development. The low relative SDA (4.7%) and minimal postprandial energetic cost suggest efficient feed processing; together with its operational and ecological advantages, this indicates probiotic-enhanced plant proteins could partially substitute fishmeal while offering economic, logistical, and ecological advantages.

Conversely, the fishmeal-based diet, despite its high-quality protein content, identical macronutrient profile, and probiotics to the plant-based formulation, induced a state of metabolic inefficiency characterized by elevated specific dynamic action (21.0%), continuous excessive nitrogen excretion, and low growth (1.49 mm/month). This outcome underscores that ingredient quality must be evaluated not only by compositional analysis but also by physiological compatibility with the target organism’s evolved digestive and metabolic capacities.

## Figures and Tables

**Table 2:** Summary of red abalone growth metrics, survival and gonadal development by treatment.

Dietary Treatment	Initial Size	Initial Weight	Final Size	Final Weight	TGs	TGw	MGRs	DGRs	MGRw	Survival	FGMI	MGMI
	mm	g	mm	g	mm	g	mm Month^−1^	µm Day^−1^	g Month^−1^	%	Day 180	Day 180
Control fresh kelp *Macrocystis pyrifera*	48.47 ± 0.45 ^a^	16.06 ± 0.54 ^a^	66.53 ± 0.50 ^a^	46.83 ± 1.14 ^a^	18.06 ± 0.67 ^a^	30.77 ± 1.26 ^a^	3.01 ± 0.11 ^a^	100.35 ± 3.73 ^a^	5.13 ± 0.21 ^a^	96.97 ± 1.72% ^a^	1.38 ± 0.08 ^a^	0.81 ± 0.05 ^a^
Plant-based + *Bacillus* FF	48.61 ± 0.47 ^a^	15.86 ± 0.55 ^a^	61.01 ± 0.50 ^b^	36.78 ± 0.98 ^b^	12.41 ± 0.69 ^b^	20.91 ± 1.12 ^b^	2.07 ± 0.11 ^b^	68.93 ± 3.81 ^b^	3.49 ± 0.19 ^b^	96.97 ± 1.72% ^a^	2.50 ± 0.09 ^b^	1.27 ± 0.14 ^b^
Fishmeal-based + *Bacillus* FF	48.45 ± 0.48 ^a^	16.11 ± 0.57 ^a^	57.42 ± 0.61 ^c^	28.98 ± 0.83 ^c^	8.97 ± 0.78 ^c^	12.87 ± 1.01 ^c^	1.49 ± 0.13 ^c^	49.82 ± 4.31 ^c^	2.14 ± 0.17 ^c^	93.94 ± 2.40% ^a^	1.02 ± 0.09 ^c^	1.05 ± 0.12 ^ab^

Note: Values are mean ± SE; Superscript letters indicate statistical differences. TGs = total growth in size; TGw = total growth in weight; MGRs = monthly growth rate in size; DGRs = daily growth rate in size; MGRw = monthly growth rate in weight; FGMI = female gonadal maturity index; MGMI = male gonadal maturity index; FF = formulated feed. The female and male gonadal maturity index values are from Olmos et al. [25].

**Table 3:** Summary of postprandial metabolic performance in red abalone by dietary treatment.

Dietary Treatment	Postprandial MO_2_	Post-Absorptive MO_2_	Postprandial AER	Post-Absorptive AER	Postprandial O:N	Post-Absorptive O:N	SDA (%)
	mg O_2_ g^−1^ Soft Tissue h^−1^	mg O_2_ g^−1^ Soft Tissue h^−1^	mg NH_3_-N g^−1^ Soft Tissue h^−1^	mg NH_3_-N g^−1^ Soft Tissue h^−1^	Atomic (95% CI)	Atomic (95% CI)	Relative Index
Control fresh kelp *Macrocystis pyrifera*	0.0127 ± 0.0003 ^a^	0.0122 ± 0.0004 ^a^	0.0041 ± 0.0005 ^a^	0.0021 ± 0.0005 ^a^	2.72 (2.17–3.52)	5.04 (3.49–8.13)	4.3 ^a^
Plant-based + *Bacillus* FF	0.0118 ± 0.0005 ^ab^	0.0113 ± 0.0004 ^ab^	0.0025 ± 0.0003 ^b^	0.0036 ± 0.0004 ^b^	4.21 (3.38–5.38)	2.73 (2.20–3.51)	4.7 ^a^
Fishmeal-based + *Bacillus* FF	0.0110 ± 0.0005 ^b^	0.0091 ± 0.0004 ^b^	0.0027 ± 0.0003 ^b^	0.0028 ± 0.0006 ^ab^	3.55 (2.82–4.53)	2.88 (1.96–4.67)	21.0 ^b^

Note: Values represent means ± SE (MO_2_, AER) or ratio-of-means with 95% bootstrap confidence interval (O:N; 10,000 resamples). Within MO_2_ and AER columns, different superscript letters indicate significant differences among diets (*p* < 0.05; linear mixed-effects models with Tukey HSD). MO_2_ = oxygen consumption rate; AER = ammonia excretion rate; SDA = relative specific dynamic action; FF = formulated feed.

## Data Availability

The original contributions presented in this study are included in the article material. Further inquiries can be directed to the corresponding author.

## Abbreviations

The following abbreviations are used in this manuscript:

AER	Ammonia excretion rate
ANF	Anti-nutritional factor
ANOVA	Analysis of variance
CFU	Colony-forming units
CICESE	Centro de Investigación Científica y Educación Superior de Ensenada
DF	Digestible fiber
DGR	Daily growth rate
DGRs	Daily growth rate in size
DOI	Digital object identifier
FF	Formulated feed
FGMI	Female gonadal maturity index
GRAS	Generally Recognized as Safe
HSD	Honestly significant difference (Tukey’s)
LME	Linear mixed-effects (model)
MGMI	Male gonadal maturity index
MGR	Monthly growth rate
MGRs	Monthly growth rate in size
MGRw	Monthly growth rate in weight
MO_2_	Oxygen consumption rate
mTOR	Mechanistic target of rapamycin
NDF	Non-digestible fiber
O:N	Oxygen-to-nitrogen ratio
SCFA	Short-chain fatty acids
SD	Standard deviation
SDA	Specific dynamic action
SE	Standard error
SECIHTI	Secretaría de Ciencia, Humanidades, Tecnología e Innovación
TGs	Total growth in size
TGw	Total growth in weight
